# Imputation of the Date of HIV Seroconversion in a Cohort of Seroprevalent Subjects: Implications for Analysis of Late HIV Diagnosis

**DOI:** 10.1155/2012/725412

**Published:** 2011-10-15

**Authors:** Paz Sobrino-Vegas, Santiago Pérez-Hoyos, Ronald Geskus, Belén Padilla, Ferrán Segura, Rafael Rubio, Jorge del Romero, Jesus Santos, Santiago Moreno, Julia del Amo

**Affiliations:** ^1^Red de Investigación en Sida, Centro Nacional de Epidemiología, Instituto de Salud Carlos III, 28029 Madrid, Spain; ^2^Institut de Recerca Hospital Vall Hebrón, Unitat Suport Metodològic a l'Investigació Biomedica, 08035 Barcelona, Spain; ^3^Cluster of Infectious Diseases, Amsterdam Public Health Service, Amsterdam, The Netherlands; ^4^Department of Clinical Epidemiology, Biostatistics and Bioinformatics, Academic Medical Centre (AMC), Amsterdam, The Netherlands; ^5^Unidad de Enfermedades Infecciosas, Hospital General Universitario Gregorio Marañón, 28007 Madrid, Spain; ^6^Corporació Sanitària Parc Taulí, 08208 Sabadell, Spain; ^7^Unidad de VIH, Hospital Universitario Doce de Octubre, 28041 Madrid, Spain; ^8^Centro Sanitario Sandoval, 28010 Madrid, Spain; ^9^Hospital Virgen de la Victoria, 29010 Málaga, Spain; ^10^Servicio de Enfermedades Infecciosas, Hospital Universitario Ramón y Cajal, 28034 Madrid, Spain

## Abstract

*Objectives*. Since subjects may have been diagnosed before cohort entry, analysis of late HIV diagnosis (LD) is usually restricted to the newly diagnosed. We estimate the magnitude and risk factors of LD in a cohort of seroprevalent individuals by imputing seroconversion dates. *Methods*. Multicenter cohort of HIV-positive subjects who were treatment naive at entry, in Spain, 2004–2008. Multiple-imputation techniques were used. Subjects with times to HIV diagnosis longer than 4.19 years were considered LD. *Results*. Median time to HIV diagnosis was 2.8 years in the whole cohort of 3,667 subjects. Factors significantly associated with LD were: male sex; Sub-Saharan African, Latin-American origin compared to Spaniards; and older age. In 2,928 newly diagnosed subjects, median time to diagnosis was 3.3 years, and LD was more common in injecting drug users. *Conclusions*. Estimates of the magnitude and risk factors of LD for the whole cohort differ from those obtained for new HIV diagnoses.

## 1. Introduction

The majority of clinical cohorts of HIV-infected people are made up of seroprevalent subjects whose dates of seroconversion are unknown [1–3]. Seroprevalent subjects have been used to quantify the magnitude and risk factors of late diagnosis of HIV infection, an important public health problem which, by definition, cannot be studied in seroconverter cohorts [4, 5]. Although there are multiple definitions of late diagnosis based on different biological markers [4, 6–8], most of them are based on the patient's CD4 lymphocyte count close to the date of HIV diagnosis. For some persons, HIV may have been diagnosed before their inclusion in a clinical cohort; therefore, no CD4 counts close to HIV diagnosis are usually available. Consequently, these people are ignored, and estimates are obtained only from those with available CD4 counts—largely the new HIV diagnoses—rather than from the whole cohort. Most clinical cohorts include newly diagnosed people as well as people who have been diagnosed in the past, but the latter group is rendered invisible. The use of multiple imputation techniques to estimate the time between HIV seroconversion and HIV diagnosis could overcome the aforementioned problem. These techniques, which so far have not been applied to study late HIV diagnosis, are based on the correlation between certain biological markers like CD4 lymphocytes and the duration of infection [9–11]. 

The magnitude of late HIV diagnoses in the subgroup of new HIV diagnoses in cohorts from industrialized countries ranges from 18% to 39% [4, 5, 12–14]. For these cohorts, the proportion of subjects who are new HIV diagnoses—and therefore can be analyzed—ranges from 4% to 73% [4, 5, 12–15]. In Spain, considering late diagnosis as subjects with a CD4 lymphocyte count of <200 cells/mm^3^ or an AIDS-defining disease in the first year after HIV diagnosis, we reported 37% of late diagnosis in 2004–06 in the 68% of subjects who could be evaluated because they were newly diagnosed at inclusion in the cohort [16]. Risk of late diagnosis increased with age, was higher in men than in women, and, contrary to previous publications [12, 17, 18], was higher in heterosexuals and injection drug users (IDUs) compared to men who have sex with men (MSM). We hypothesized that this unexpected finding may reflect that the new diagnoses represent a different population than the old ones, which could not be evaluated for late diagnosis analyses [16]. To test this hypothesis, we estimated the magnitude and risk factors of late HIV diagnosis, in all cohort members and separately in those newly diagnosed, in a multicenter cohort of seroprevalent subjects in Spain for whom we have imputed their HIV seroconversion dates.

## 2. Methods

CoRIS is an open, multicenter, and prospective cohort of adult patients with confirmed HIV infection who are naive to antiretroviral treatment (ART) at the first visit to any of the CoRIS centers and who agree to participate in the study by signing an informed consent form. A complete description has been published elsewhere [19]. Briefly, CoRIS collects a minimum dataset which is subject to internal and external quality controls. Between January 2004 and October 2008, 4,057 subjects were recruited from 27 participating centers where the percentage of CD4 lymphocytes (hereinafter referred to as “CD4%”) was measured. A total of 231 subjects were excluded because they had recently been recruited, and no CD4% results were available, and 159 were excluded because their first CD4% values were recorded after ART initiation. Accordingly, 3,667 patients were available for analysis. 

Subjects were classified as late diagnosis (LD) when the diagnosis of HIV infection was made more than 4.19 years after seroconversion. This cut-off point was chosen because, in a previous publication [20], it was estimated that this was the time elapsed from seroconversion to reaching a CD4 threshold of <350. In turn, this CD4 lymphocyte threshold is used in the new definition of late presentation recommended by the European Late Presenter Consensus Working Group [6]. 

A multiple imputation technique was used to estimate the date of seroconversion of all CoRIS subjects, based on the model for progression of infection described by Muñoz et al. [10], which has been used in Spain [11]. These authors use parametric survival models based on the Weibull distribution to estimate the time elapsed between the date of HIV seroconversion and the date of first CD4% in the absence of ART, on the basis of that first CD4%. Their paper describes the model's parameter for each of the five thresholds in which CD4% is categorized. 

This model and its coefficients allow us to know the probability that the date of seroconversion falls before a given date, conditioned by the fact that it must be between the date when the subject started being at risk for HIV infection and the date of HIV diagnosis. From this model equation, we can estimate (impute) the timespan between the date of seroconversion and the date of HIV diagnosis when the following information is made available for each subject: (a) date when the subject started being at risk for HIV infection, (b) date of HIV diagnosis, and (c) the value of CD4% and the date it was measured. 

We used the following imputation process: (1) for each individual, a random number was drawn from a Weibull distribution with the parameters corresponding to his/her CD4% threshold, which was considered a random estimate of the timespan between the date of seroconversion and the date of first CD4% (*t*). This made it possible to calculate the timespan between the date of seroconversion and the date of HIV diagnosis (“time to HIV diagnosis”, *t_1_*), and the date of seroconversion as the difference between the date of first CD4% minus time *t*. Subjects whose time *t_1_* was longer than 4.19 years were considered late diagnoses. (2) The preceding process was replicated 20 times. Twenty different databases were generated with the information obtained in each replication. (3) The subsequent analyses were made by combining the results obtained when analyzing these 20 databases separately. 

We also present the results obtained using the definition that classified subjects as delayed diagnosis (DD) when they had a CD4 lymphocyte count of <350 cells/mm^3^ in the first year after HIV diagnosis or an AIDS-defining disease in the first three months after HIV diagnosis. Thus, this definition only permitted the evaluation of subjects for whom that information was available, that is, the new HIV diagnoses.

We assumed that the date when a subject started being at risk for HIV infection was the beginning of the epidemic in Spain, 1 January 1980, except in (a) patients infected by the sexual route or by injecting drug use who were born after 1 January 1965; for these subjects, we used the date of their 15th birthday, and (b) patients in the remaining transmission categories who were born after 1 January 1980, for whom we used their date of birth.

We present a descriptive analysis of the characteristics of subjects included in the analysis, as well as their time to HIV diagnosis. We used an analysis of variance for the comparison of means, to compare the time to HIV diagnosis according to patient characteristics.

To evaluate the factors independently associated with late diagnosis, we used a multivariate logistic regression model. In this model, robust methods were used to estimate the confidence intervals, assuming correlation among subjects recruited in each center and independence between subjects in different centers [21].

The analyses were performed using R version 2.13 [22] and Stata 11.

## 3. Results

Of the 3,667 patients included in this analysis, most were men (77.8%), were infected by sexual transmission (43.1% MSM and 37.5% heterosexual), and were Spanish nationals (68.5%); 15.8% had been infected through injecting drug use. The mean age at HIV diagnosis was 34.8 years (SD = 10.2) and the median follow-up time was 1.38 years. At cohort entry, 442 patients (12%) had been diagnosed with AIDS, another 191 (5.2%) developed AIDS, and 86 persons (2.3%) died during followup.

### 3.1. Description of Time from Imputed Seroconversion to HIV Diagnosis

#### 3.1.1. Results for All Subjects Included in the Cohort (*n* = 3667)

**Figure 1:**
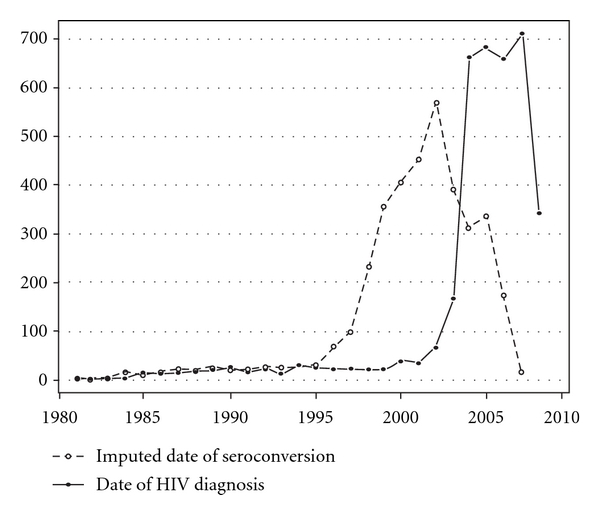
Distribution of date of seroconversion and date of HIV diagnosis.

The distribution of the dates of HIV diagnosis and the mean imputed seroconversion date per individual can be seen in Figure 1. The shape of the figure is similar in both cases, but with a shift over time. The median date of HIV diagnosis was October 2005 (IQR: June 2004–February 2007) while the median date of seroconversion was February 2002 (IQR: May 1999–May 2004). 

Table 1 shows the distribution of years elapsed between the mean imputed date of seroconversion and the date of HIV diagnosis. Overall, the median time to HIV diagnosis was 2.8 years (IQR: 1.2–5.2).

Time to HIV diagnosis was longer in men, in persons with heterosexual or “other” routes of transmission (vertical, transfusions, tattoos,…), and, in those from countries other than Spain, it also increased with age at HIV diagnosis and was longer in patients who developed AIDS and in those who died.

Table 2 shows the distribution of late diagnosis according to the sociodemographic characteristics of the subjects and the odds ratio based on the multivariate analysis. Factors independently associated with late diagnosis in the multivariate analysis were male gender, place of origin Sub-Saharan Africa or Latin America, and older age at HIV diagnosis. Subjects with heterosexual transmission had a higher frequency of late diagnoses than MSM although that higher frequency did not attain statistical significance.

#### 3.1.2. Results in the Subgroup of New HIV Diagnoses (*n* = 2928)

In this subgroup of new HIV diagnoses (*n* = 2,928), the median time to HIV diagnosis was 3.3 years (IQR: 1.6–5.7) (Table 3), longer than the median of 2.8 years estimated for the whole cohort.

These differences can partly be explained by the fact that the 739 subjects excluded from the analyses were significantly different (*P* < 0.05) from the 2,928 who were included; in the following ways, they were younger at diagnosis (mean age 30 versus 36 years) and at seroconversion (mean age 28 versus 32 years); they were more frequently IDUs (38.2% versus 10.1%); they were more often of Spanish origin (75.0% versus 66.9%). 

Table 2 shows the distribution of late diagnosis and the results of the multivariate analysis in this subcohort. Unlike what was seen in the whole cohort of 3,667 subjects, IDUs had a higher frequency of late diagnoses compared with MSM. Subjects with heterosexual transmission also had a significantly higher frequency of late diagnoses than MSM.

With regard to sex, age of diagnosis, and country of origin, the results were similar to those for the whole cohort.

Table 3 shows the estimated time to HIV diagnosis in this group and the percentage of delayed diagnoses according to the definition DD. For each of the sociodemographic characteristics studied in the subgroup of 2,928 new HIV diagnoses, we observed high consistency, except in women, between time from imputed seroconversion date to HIV diagnosis and frequency of delayed diagnoses (DD).

## 4. Discussion

This study illustrates the application of a multiple imputation method to estimate the date of HIV seroconversion in a cohort of seroprevalent patients who are not all newly diagnosed with HIV at entry. We defined as late diagnosis the subjects with times to HIV diagnosis longer than 4.19 years. The advantage of this definition is that it allows estimation of late diagnosis in the whole cohort and not just in patients with CD4 markers close to the time of HIV diagnosis. 

Half of the cohort members were not diagnosed with HIV until 2.8 years after becoming infected, and one fourth were not diagnosed until 5.2 years after infection. Based on the multivariate analysis, the time between the imputed date of HIV seroconversion and HIV diagnosis was longer in men, increased with age, and was longer in persons from Sub-Saharan Africa and Latin America compared to Spaniards. In contrast, half of the new HIV diagnoses at entry into the cohort were not diagnosed until 3.3 years after their imputed HIV seroconversion date, and diagnostic delay was more common in IDUs. 

By imputing the date of seroconversion, we have shown that the magnitude of late diagnosis in the whole cohort was smaller than in the subgroup of new diagnoses (34% versus 39%). In addition, we found differences not only in the magnitude of late diagnosis but also in the associated risk factors. These differences reflect the important changes in HIV epidemiology, and probably in HIV testing practices as well, that have taken place in Spain in the last decade: a major reduction in the number of IDUs who were exposed to frequent HIV testing opportunities, together with an increase in sexually acquired infections which continues to require more active HIV testing approaches. As CoRIS is not population based, these conclusions cannot be extrapolated to the whole HIV-positive population in Spain.

Our group had already evaluated late diagnosis in the cohort, but limited to those patients with an HIV diagnosis close to the time of their inclusion in the cohort [16]. We had observed a very high prevalence of late diagnoses in IDUs, a result that differed from other studies carried out in Spain which described very high HIV testing uptake in IDUs [12, 17, 18]. Here, by imputing the date of seroconversion, which allows study of the whole cohort, we no longer see a higher frequency of late diagnoses in IDUs although this pattern continues to be seen in the subgroup of new diagnoses. What this reflects is that IDUs diagnosed with HIV before cohort entry—in drug attention centers—were excluded from the analyses. Together with a marked decline in the number of IDUs among new HIV diagnoses in Spain, the analyses of late HIV diagnosis within the surveillance system have also identified a higher frequency of late diagnosis among IDUs [23]. Consistent with previous publications from Spain and other countries [12, 13, 16, 17, 23], late diagnosis is higher in men, in migrants from non-Western countries, and increases with age.

The results of this study are also important for comparison purposes as the proportion of new HIV diagnoses in a cohort may vary between cohorts and within the same cohort over time. For example, cohorts may increase the number of recruiting sites, or HIV incidence may change in a given group. In this work, we highlight the fact that new HIV diagnoses do not represent the whole cohort and that their relative contribution needs to be taken into account when comparing different cohorts or when interpreting trends over time. 

Our results are based on imputing the date of seroconversion by using the first available CD4 percentage from each patient while off treatment. Other authors have observed that this estimate can be improved by using the evolution of various CD4 measurements [24, 25]. We also performed this imputation process for each measurement of CD4 percentage and estimated the date of seroconversion as the median date of seroconversion estimated by the imputation for each value of CD4. No differences were found with this analysis; the median date of seroconversion was 1 July 2002. This may be because the median number of CD4 measurements in persons off treatment was only two, since most people start treatment soon after entry. 

We also conducted several sensitivity analyses using different assumptions about the date of initial risk, and the results were similar. 

To evaluate the influence of the distribution model initially selected to impute the date of seroconversion [10], we analyzed the data based on Weibull models with different parameters which, in some cases, permitted a subject to have been infected for 30 years at the time of CD4 measurement. The results obtained did not differ substantially from those presented.

Time to HIV diagnosis and delayed diagnosis (DD) was not highly consistent in women. Some studies have shown that, after seroconversion, women take longer than men to reach the same CD4 level [20, 26]. Lodi et al. estimate these differences at between 6 and 12 months [20]. We conducted an analysis considering for women a Weibull distribution with the same shape parameter, but with a median of 9 months longer than for men. In this simulation, differences between men and women in time to diagnosis and in the percentage of late diagnosis disappear.

In conclusion, estimates of the magnitude and risk factors of late HIV diagnoses for an entire cohort may differ from those obtained for new HIV diagnoses, a finding that highlights the need to both improve and expand HIV testing practices in our setting.

## Figures and Tables

**Table 1:** Years between imputed date of seroconversion and date of HIV diagnosis in the whole cohort (*n* = 3667).

	Years between imputed date of seroconversion and date of HIV diagnosis			
	*N*	Median (P_25_–P_75_)	Mean	*P*
*Sex*				<0.001
Men	2854	2.90 (1.24–5.40)	3.66	
Women	813	2.32 (0.98–4.60)	3.15	

*Age at HIV diagnosis*				<0.001
Up to 20	158	1.31 (0.49–2.55)	1.66	
21–30	1239	2.11 (0.86–4.36)	2.93	
31–40	1361	2.99 (1.32–5.56)	3.78	
41–50	599	3.67 (1.70–6.05)	4.25	
51–60	219	4.16 (2.18–6.44)	4.59	
Over 60	74	4.27 (2.22–6.42)	4.57	
Not available	17	3.66 (2.04–5.57)	4.11	—

*Transmission category*				<0.001
Injection drug user	578	1.82 (0.59–4.40)	2.87	
Men who have sex with men	1581	2.73 (1.25–5.17)	3.57	
Heterosexual risk exposure	1376	3.08 (1.38–5.53)	3.76	
Other (vertical, transfusions, tattoos, etc.)	58	3.03 (1.33–5.48)	3.63	
Don't know/No answer	74	3.81 (1.83–6.10)	4.28	

*Educational level*				0.790
No education or less than primary	257	2.88 (1.07–5.20)	3.46	
Primary	1214	2.68 (1.09–5.20)	3.49	
Secondary completed	1011	2.79 (1.22–5.25)	3.57	
University completed	524	2.84 (1.29–5.33)	3.66	
Not available	661	2.75 (1.19–5.20)	3.54	—

*Country of origin*				0.011
Spain	2512	2.57 (1.07–5.06)	3.42	
Western Europe	114	2.96 (1.23–5.40)	3.74	
Eastern Europe and Russia	72	2.97 (1.46–5.16)	3.58	
Sub-Saharan Africa	246	3.54 (1.68–5.88)	4.04	
North Africa	58	3.89 (1.77–6.35)	4.51	
Latin America	631	3.04 (1.36–5.45)	3.71	
Other/not available	34	2.84 (1.46–5.25)	3.77	

*AIDS*				<0.001
Yes	633	4.13 (2.01–6.19)	4.32	
No	3034	2.49 (1.09–4.90)	3.38	

*Death*				0.081
Yes	86	4.08 (1.84–6.12)	4.25	
No	3581	2.73 (1.16–5.21)	3.53	

*Total*	3667	2.76 (1.17–5.24)	3.54	

**Table 2:** Factors associated with late diagnosis (time to HIV diagnosis over 4.19 years).

	Whole cohort (*N* = 3667)				New HIV diagnoses (*N* = 2928)			
	LD*/Total	%	Adjusted OR (95% CI)	*P*	LD*/Total	%	Adjusted OR (95% CI)	*P*
*Sex*								
Men	1018/2854	36	1.67 (1.27–2.21)	<0.001	936/2287	41	1.77 (1.30–2.40)	<0.001
Women	233/813	29	1		211/641	33	1	

*Age at HIV diagnosis*				<0.001				<0.001
Up to 30	337/1397	24	1		296/964	31	1	
31–40	498/1361	37	1.79 (1.46–2.19)	<0.001	459/1133	41	1.49 (1.19–1.87)	<0.001
Over 41	408/892	46	2.64 (2.10–3.30)	<0.001	392/830	47	1.95 (1.56–2.45)	<0.001
Not available	7/17	42	1.99 (0.61–6.54)	0.256	1/1	—	—	0.808

*Transmission category*				0.055				0.043
Injection drug user	154/578	27	0.83 (0.62–1.09)	0.176	136/296	46	1.60 (1.10–2.32)	0.014
Men who have sex with men	526/1581	33	1		479/1339	36	1	
Heterosexual risk exposure	516/1376	37	1.26 (0.98–1.62)	0.069	483/1187	41	1.39 (1.06–1.82)	0.016
Other (vertical, transfusions, tattoos, etc.)	21/58	37	1.08 (0.56–2.09)	0.820	20/46	44	1.36 (0.68–2.72)	0.385
Don't know/No answer	34/74	45	1.47 (0.77–2.79)	0.241	30/60	50	1.70 (0.89–3.21)	0.105

*Country of origin*				0.004				0.065
Spain	812/2512	32	1		743/1958	38	1	
Western Europe	41/114	36	1.14 (0.68–1.93)	0.615	34/82	41	1.11 (0.59–2.09)	0.744
Eastern Europe and Russia	25/72	35	1.48 (0.77–2.86)	0.243	23/64	37	1.13 (0.56–2.29)	0.731
Sub-Saharan Africa	104/246	42	1.75 (1.20–2.56)	0.004	95/215	44	1.54 (1.04–2.29)	0.032
North Africa	27/58	47	1.75 (0.85–3.59)	0.129	27/56	49	1.49 (0.70–3.14)	0.297
Latin America	231/631	37	1.38 (1.09–1.74)	0.008	214/522	41	1.38 (1.08–1.75)	0.009
Other/not available	11/34	34	0.96 (0.39–2.34)	0.925	11/31	36	0.92 (0.39–2.18)	0.846

*Total*	1251/3667	34			1147/2928	39		

*LD.: Late diagnosis (patients with time to HIV diagnosis over 4.19 years).

**Table 3:** Years between the imputed date of seroconversion and the date of HIV diagnosis. Results in the subcohort of new diagnoses (*n* = 2928).

	Years between imputed date of seroconversion and date of HIV diagnosis				
	*N*	% DD*	Median (P_25_–P_75_)	Mean	*P*
*Sex*					<0.001
Men	2287	51.8	3.42 (1.65–5.83)	4.07	
Women	641	50.2	2.77 (1.32–5.04)	3.51	

*Age at HIV diagnosis *					<0.001
Up to 20	93	33.3	1.94 (1.08–3.05)	2.15	
21–30	871	39.6	2.76 (1.33–5.01)	3.46	
31–40	1133	52.0	3.36 (1.62–5.89)	4.09	
41–50	549	61.9	3.83 (1.87–6.18)	4.38	
51–60	209	69.9	4.22 (2.25–6.42)	4.60	
Over 60	72	75.0	4.37 (2.29–6.47)	4.63	
Not available	1	100.0	5.25 (5.25–5.25)	5.25	—

*Transmission category*					0.111
Injection drug user	296	66.6	3.86 (1.93–6.11)	4.34	
Men who have sex with men	1339	41.3	2.97 (1.42–5.43)	3.77	
Heterosexual risk exposure	1187	58.0	3.39 (1.62–5.79)	4.00	
Other (vertical, transfusions, tattoos, etc.)	46	63.0	3.71 (2.02–5.94)	4.18	
Don't know/No answer	60	63.3	4.18 (2.12–6.44)	4.57	

*Educational level*					0.640
No education or less than primary	187	63.1	3.62 (1.77–5.85)	4.06	
Primary	928	57.0	3.40 (1.62–5.82)	4.05	
Secondary completed	853	46.0	3.13 (1.48–5.57)	3.85	
University completed	443	41.3	3.07 (1.48–5.55)	3.85	
Not available	517	54.9	3.30 (1.61–5.61)	3.96	—

*Country of origin*					0.410
Spain	1958	49.4	3.14 (1.49–5.58)	3.87	
Western Europe	82	50.0	3.47 (1.77–5.80)	4.19	
Eastern Europe and Russia	64	51.6	3.15 (1.59–5.33)	3.74	
Sub-Saharan Africa	215	61.9	3.73 (1.82–6.05)	4.21	
North Africa	56	60.7	4.09 (1.88–6.49)	4.62	
Latin America	522	54.8	3.43 (1.70–5.81)	4.04	
Other/not available	31	38.7	3.09 (1.52–5.73)	3.97	

*All*	2928	51.4	3.26 (1.56–5.68)	3.94	

*DD: Delayed diagnosis (patients with CD4 count <350 cells/mm^3^ in the first year after HIV diagnosis or with AIDS-defining disease in the the first three months after HIV diagnosis).

## Appendix

### Centers and Investigators Participatingin CoRIS

1. Coordinating Committee Juan Berenguer, Julia del Amo, Federico García, Félix Gutiérrez, Pablo Labarga, Santiago Moreno y María Ángeles Muñoz.

2. Field Work, Data Management and Analysis Ana María Caro-Murillo, Paz Sobrino Vegas, Santiago Pérez-Cachafeiro, Victoria Hernando Sebastián, Belén Alejos Ferreras, Débora Álvarez, Susana Monge, Inma Jarrín, Mónica Trastoy.

3. BioBanco M Ángeles Muñoz-Fernández, Isabel García-Merino, Coral Gómez Rico, Jorge Gallego de la Fuente y Almudena García Torre.

4. Participating Centers
Hospital General Universitario de Alicante (Alicante) Joaquín Portilla Sogorb, Esperanza Merino de Lucas, Sergio Reus Bañuls, Vicente Boix Martínez, Livia Giner Oncina, Carmen Gadea Pastor, Irene Portilla Tamarit, Patricia Arcaina Toledo.
Hospital Universitario de Canarias (Santa Cruz de Tenerife) Juan Luis Gómez Sirvent, Patricia Rodríguez Fortúnez, María Remedios Alemán Valls, María del Mar Alonso Socas, Ana María López Lirola, María Inmaculada Hernández Hernández, Felicitas Díaz-Flores.
Hospital Carlos III (Madrid) Vicente Soriano, Pablo Labarga, Pablo Barreiro, Carol Castañares, Pablo Rivas, Andrés Ruiz, Francisco Blanco, Pilar García, Mercedes de Diego.
Hospital Universitario Central de Asturias (Oviedo) Victor Asensi, Eulalia Valle, José Antonio Cartón.
Hospital Clinic (Barcelona) José M. Miró, María López-Dieguez, Christian Manzardo, Laura Zamora, Iñaki Pérez, M*ª* Teresa García, Carmen Ligero, José Luis Blanco, Felipe García-Alcaide, Esteban Martínez, Josep Mallolas, José M. Gatell.
Hospital Doce de Octubre (Madrid) Rafael Rubio, Federico Pulido, Silvana Fiorante, Jara Llenas, Violeta Rodríguez, Mariano Matarranz.
Hospital Donostia (San Sebastián) José Antonio Iribarren, Julio Arrizabalaga, María José Aramburu, Xabier Camino, Francisco Rodríguez-Arrondo, Miguel Ángel von Wichmann, Lidia Pascual Tomé, Miguel Ángel Goenaga, M*ª* Jesús Bustinduy, Harkaitz Azkune Galparsoro.
Hospital General Universitario de Elche (Elche) Félix Gutiérrez, Mar Masiá, José Manuel Ramos, Sergio Padilla, Andrés Navarro, Fernando Montolio, Yolanda Peral, Catalina Robledano García.
Hospital Germans Trías i Pujol (Badalona) Bonaventura Clotet, Cristina Tural, Lidia Ruiz, Cristina Miranda, Roberto Muga, Jordi Tor, Arantza Sanvisens.
Hospital Gregorio Marañón (Madrid) Juan Berenguer, Juan Carlos López Bernaldo de Quirós, Pilar Miralles, Jaime Cosín Ochaíta, Matilde Sánchez Conde, Isabel Gutiérrez Cuellar, Margarita Ramírez Schacke, Belén Padilla Ortega, Paloma Gijón Vidaurreta.
Hospital Universitari de Tarragona Joan XXIII, IISPV, Universitat Rovira i Virgili (Tarragona) Francesc Vidal, Joaquín Peraire, Consuelo Viladés, Sergio Veloso, Montserrat Vargas, Miguel López-Dupla, Montserrat Olona, Joan-Josep Sirvent, Alba Aguilar, Antoni Soriano.
Hospital Universitario La Fe (Valencia) José López Aldeguer, Marino Blanes Juliá, José Lacruz Rodrigo, Miguel Salavert, Marta Montero, Eva Calabuig, Sandra Cuéllar.
Hospital Universitário La Paz (Madrid) Juan González García, Ignacio Bernardino de la Serna, José María Peña Sánchez de Rivera, Marta Mora Rillo, José Ramón Arribas López, María Luisa Montes Ramírez, José Francisco Pascual Pareja, Blanca Arribas, Juan Miguel Castro, Fco Javier Zamora Vargas, Ignacio Pérez Valero.
Hospital de la Princesa (Madrid) Ignacio de los Santos, Jesús Sanz Sanz, Johana Rodríguez, Ana Salas Aparicio, Cristina Sarriá Cepeda.
Hospital San Pedro-CIBIR (Logroño) José Antonio Oteo, José Ramón Blanco, Valvanera Ibarra, Luis Metola, Mercedes Sanz, Laura Pérez-Martínez.
Hospital San Pedro II (Logroño) Javier Pinilla Moraza.
Hospital Universitario Mutua de Terrassa (Terrassa) David Dalmau, Angels Jaén Manzanera, Mireia Cairó Llobell, Daniel Irigoyen Puig, Laura Ibáñez, Queralt Jordano Montañez, Mariona Xercavins Valls, Javier Martinez-Lacasa, Pablo Velli, Roser Font.
Hospital de Navarra (Pamplona) Julio Sola Boneta, Javier Uriz, Jesús Castiello, Jesús Reparaz, María Jesús Arraiza, Carmen Irigoyen, David Mozas.
Hospital Parc Taulí (Sabadell) Ferrán Segura, María José Amengual, Eva Penelo, Gemma Navarro, Montserrat Sala, Manuel Cervantes, Valentín Pineda.
Hospital Ramón y Cajal (Madrid) Santiago Moreno, José Luis Casado, Fernando Dronda, Ana Moreno, María Jesús Pérez Elías, Dolores López, Carolina Gutiérrez, Beatriz Hernández, María Pumares, Paloma Martí.
Hospital Reina Sofía (Murcia) Alfredo Cano Sánchez, Enrique Bernal Morell, Ángeles Muñoz Pérez.
Hospital San Cecilio (Granada) Federico García García, José Hernández Quero, Alejandro Peña Monje, Leopoldo Muñoz Medina, Jorge Parra Ruiz.
Centro Sanitario Sandoval (Madrid) Jorge Del Romero Guerrero, Carmen Rodríguez Martín, Teresa Puerta López, Juan Carlos Carrió Montiel, Cristina González.
Hospital Universitario Santiago de Compostela (Santiago de Compostela) Antonio Antela, Arturo Prieto, Elena Losada.
Hospital Son Dureta (Palma de Mallorca) Melchor Riera, Javier Murillas, Maria Peñaranda, Maria Leyes, M*ª* Angels Ribas, Antoni Campins, Concepcion Villalonga.
Hospital Universitario de Valme (Sevilla) Juan Antonio Pineda, Eva Recio Sánchez, Fernando Lozano de León, Juan Macías, José del Valle, Jesús Gómez-Mateos, Rosario Mata.
Hospital Virgen de la Victoria (Málaga) Jesús Santos González, Manuel Márquez Solero, Isabel Viciana Ramos, Rosario Palacios Muñoz.
Hospital Universitario Virgen del Rocío (Sevilla) Pompeyo Viciana, Manuel Leal, Luis Fernando López-Cortés, Mónica Trastoy.
